# Effects of Behavioral Risk Factors and Social-Environmental Factors on Non-Communicable Diseases in South Korea: A National Survey Approach

**DOI:** 10.3390/ijerph18020612

**Published:** 2021-01-12

**Authors:** Nam Jeong Jeong, Eunil Park, Angel P. del Pobil

**Affiliations:** 1 Department of Interaction Science, College of Computing, Sungkyunkwan University, Seoul 03063, Korea; skawjdzz@skku.edu; 2 Robotic Intelligence Laboratory, Engineering and Computer Science Deparment, University Jaume-I, 12071 Castellon, Spain

**Keywords:** non-communicable diseases, behavioral risk factors, social-environmental factors, path analysis

## Abstract

Non-communicable diseases (NCDs) are one of the major health threats in the world. Thus, identifying the factors that influence NCDs is crucial to monitor and manage diseases. This study investigates the effects of social-environmental and behavioral risk factors on NCDs as well as the effects of social-environmental factors on behavioral risk factors using an integrated research model. This study used a dataset from the 2017 Korea National Health and Nutrition Examination Survey. After filtering incomplete responses, 5462 valid responses remained. Items including one’s social-environmental factors (household income, education level, and region), behavioral factors (alcohol use, tobacco use, and physical activity), and NCDs histories were used for analyses. To develop a comprehensive index of each factor that allows comparison between different concepts, the researchers assigned scores to indicators of the factors and calculated a ratio of the scores. A series of path analyses were conducted to determine the extent of relationships among NCDs and risk factors. The results showed that social-environmental factors have notable effects on stroke, myocardial infarction, angina, diabetes, and gastric, liver, colon, lung, and thyroid cancers. The results indicate that the effects of social-environmental and behavioral risk factors on NCDs vary across the different types of diseases. The effects of social-environmental factors and behavioral risk factors significantly affected NCDs. However, the effect of social-environmental factors on behavioral risk factors was not supported. Furthermore, social-environmental factors and behavioral risk factors affect NCDs in a similar way. However, the effects of behavioral risk factors were smaller than those of social-environmental factors. The current research suggests taking a comprehensive view of risk factors to further understand the antecedents of NCDs in South Korea.

## 1. Background

Non-communicable diseases (NCDs) have been highlighted as a significant global health threat. According to the World Health Organization’s (WHO) report, NCDs accounted for approximately 71% of global deaths in 2016. The four major types of NCDs—cardiovascular disease (CVD), cancer, diabetes, and chronic pulmonary disease—are responsible for the majority of deaths by NCDs [1].

The South Korean government reported that over 60% of deaths in 2017 were significantly associated with NCDs [2]. Among them, cancer, CVD, and diabetes were the top three causes of NCD deaths, accounting for 27.6%, 20.8%, and 17.9%, respectively [2].

Prior studies on NCDs indicated that both environmental and behavioral risk factors play critical roles in the development of NCDs [3,4]. The environment can be divided into physical and social dimensions [5]. The social environment includes one’s social belonging, neighborhood, and overall circumstances [5], while it determines several indicators of NCDs (e.g., mortality, incidence, or prevalence) [6].

In the case of behavioral risk factors, the WHO reports that tobacco use, physical inactivity, unhealthy diet, and harmful use of alcohol are four major determinants of NCDs [7]. The WHO indicates that all these behavioral risk factors have considerable effects on NCDs. For instance, tobacco use accounts for nearly 6 million deaths worldwide. Both physical inactivity and harmful use of alcohol account for about 3.2 million and 2.3 million deaths, respectively [3].

In an attempt to reduce the global incidence rate of NCDs, the WHO presented “*the Global Action Plan for the Prevention and Control of NCDs*” [7]. The action plan provides instructions for managing major behavioral risk issues, which are defined as common modifiable risk factors, through local and national healthcare systems.

NCDs are conceptualized as chronic diseases in South Korea [8]. In addition to their continually growing threat to national health and other medical areas, the government has strengthened healthcare policies to decrease the burden of NCDs [8]. For example, the government expanded the coverage of national insurance plans to include four major disease categories: cancer, heart diseases, cerebrovascular disease, and rare or intractable diseases [9].

However, concerning the fact that the national plans and policies in South Korea generally focus on treating illnesses rather than preventing them, attention to monitoring and preventing NCDs is needed [10,11]. Thus, the current study aims to explore the effects of social-environmental and behavioral risk factors on NCDs in South Korea, such as stroke, myocardial infarction (MI), diabetes, and cancer.

### 1.1. Social-Environmental Factors

The environment includes not only global environmental changes such as urbanization and the aging population but also the social and physical environments surrounding individuals [12]. Since the 1980s, the relationship between an individual’s social environment and their health has been extensively studied [6]. The importance of social-environmental factors’ effect on the population’s health has been elaborated by epidemiological studies [13,14]. This line of study highlights the role of social-environmental factors such as socioeconomic status and social support as the determinants of health. The studies have discovered that low socioeconomic status leads to poor health outcomes and even raises the possibility of deaths [13]. Social status works as a fundamental cause of disease as it is linked tightly to the resources such as money, knowledge, and social supports [15]. Individuals in higher social status get more benefits from the resources, thus gain positive health outcomes [13,15].

As noted in several previous studies [6,16], the social environment has notable effects on the social standing of individuals and shows a consistent correlation with individual health. This correlation means that an individual’s health status can be predicted if their social standing is known.

Over the years, the relationship between NCDs and social-environmental factors has also been widely addressed. Grotto et al. (2008) argued that lower social status is associated with higher blood pressure, based on 22 research articles on the connection between hypertension and the social environment [17]. Moreover, Dinca-Panaitescu et al. (2011) analyzed the 2005 Canadian Community Health Survey and found strong ties between an individual’s income and prevalence of type II diabetes [18].

Preceding studies on the effects of social-environmental factors on cancer survival also indicated that low-income and poorly educated populations have a lower survival rate than other populations [19,20]. However, a recent study found that the relationship between social-environmental factors and incidence of cancer can vary by cancer type or ethnic group. Based on cancer diagnosis data from California from 1998 to 2002, Yin et al. (2010) found that a more significant number of individual incidence rates of female breast cancer and prostate cancer are triggered by higher social status. While, the relationship between social status and incidence rates of other cancer types, such as colorectal, cervical, and lung cancers, varies across races and ethnic groups [21].

In another example, Robert et al. (2004) found a notable association between social-environmental factors and female breast cancer. They investigated the effects of community-level social status and living areas on developing female breast cancer based on the breast cancer case-control study in Wisconsin between 1988 and 1995 [22]. Four indices of community-level social status, including family income and community education levels, were measured while urban or rural areas categorized the living areas. The results showed that individuals with higher community-level social status and living in urban areas are more likely to be associated with breast cancer than those with lower community-level social status and living in rural areas.

Thus, based on the findings of the previous studies mentioned above on social-environmental factors, this study proposes the following hypothesis:

**Hypothesis** **1** **(H1).** *Social-environmental factors will directly affect NCDs.*

### 1.2. Behavioral Risk Factors

#### 1.2.1. Alcohol Use

There are mixed findings on the relationship between alcohol use and NCD risk. Hong et al. (2015) used the dataset of the 2017 Korea National Health and Nutrition Examination Survey (KNHANES) from 2010 to 2012 and found that excessive alcohol consumption is related to a higher risk of diabetes in South Korean males [23]. Xi et al. (2017) claimed that light or moderate alcohol intake decreases the potential risk of CVD mortality by introducing a J-shaped curve phenomenon, based on the United States (US) National Health Interview Surveys from 1997 to 2009 and the US National Death Index Records in 2011 [24].

Meanwhile, based on 156 research articles on alcohol use, Corrao et al. (2004) argued that the potential risk of developing major NCDs, including hypertension and cancer, increases due to moderate alcohol use [25]. Most prior studies also indicated that heavy or binge drinking leads to an increased risk of developing NCDs [25,26].

#### 1.2.2. Tobacco Use

Using the South Korean National Cohort Data from 1992 to 2006, Jee et al. (2010) explored the effects of tobacco use on diabetes incidence and mortality rates in South Korean adults (aged 30–95 years) [27]. Using the Cox proportional hazards model, they found that smoking contributes to increased incidence and mortality rates of diabetes.

Further, several studies identified increased cancer risks due to consistent smoking behavior [28,29,30]. Inoue-Choi and colleagues investigated the dataset of the National Institutes of Health—American Association of Retired Persons Diet and Health Study in 2004 and 2005 conducted by the National Cancer Institute and found that smoking 1–10 cigarettes per day over a lifetime leads to a more than twofold increase in smoking-related cancer risk compared to non-smokers [30]. Thus, based on the findings of previous studies on the effects of smoking behavior, the current study assumed that tobacco use is related to the incidence of NCDs.

#### 1.2.3. Physical Inactivity

The relationship between physical activity and health outcomes has been extensively examined. Morris and Crawford (1958) found a meaningful connection between physical activity at work and coronary heart diseases in the 1950s based on the National Necropsy Survey in England [31]. Specifically, they found that men with physically inactive jobs tend to be more prone to coronary heart disease. Recent research has pointed out that different physical activities significantly affect various health outcomes [32]. Based on the Third National Health and Nutrition Examination Survey dataset in the US, leisure-time physical activities were negatively associated with all-cause mortality. In contrast, occupational, physical activities were positively associated with all-cause mortality in Mexican-American workers [32].

As presented above, both social-environmental and behavioral risk factors have been recognized as significant determinants of health status. Thus, many scholars have attempted to identify the relationship between these factors [33,34,35,36]. However, even though the relationships among the factors have been investigated, the possibility of societal and temporal variations of the relationships underscores the empirical testing on different temporal and spatial contexts [35].

Therefore, based on the findings of previous studies on behavioral risk factors, the current study proposes the following hypotheses:

**Hypothesis** **2** **(H2).** *Behavioral risk factors will directly affect NCDs.*

**Hypothesis** **3** **(H3).** *Social-environmental factors will directly affect behavioral risk factors.*

**Figure 1:**
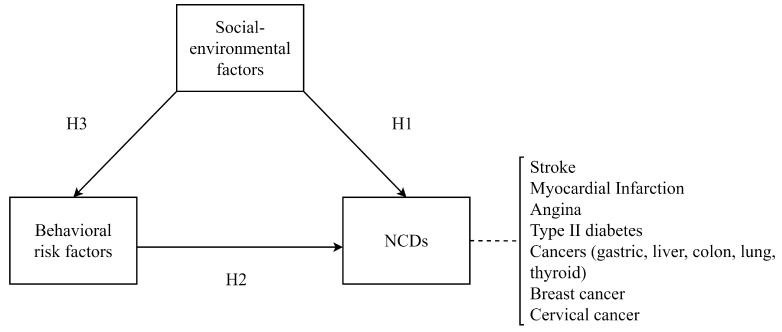
The research model.

To investigate the hypotheses, we constructed the following research model (Figure 1).

## 2. Method

### 2.1. Data Description

We utilized the 2017 dataset of the KNHANES for this study [37,38]. Since 1998, the Korea Centers for Disease Control and Prevention (KCDC) has conducted the KNHANES to monitor the general health and nutritional status. The survey used interviews, examinations, and nutrition questionnaire items, including socio-demographic information, health and biochemical status, health-related behaviors, and dietary habits. Both the interviews and examinations were carried out by professional medical staff. In addition, whole survey procedures were conducted with the respondents’ consent. The anonymized dataset of 2017 KNHANES was published in January 2019 and offers public access.

### 2.2. Sample

All respondents were South Korean. The sample for the survey was extracted using multi-stage clustering probability sampling based on the 2010 Population and Housing Census, with a sample size of 10,430 [38]. Those who responded to at least one of the interviews were 8128 (77.9%). Of the 8128 responses, 6519 (80.2%) were adults. After filtering the responses (e.g., excluding incomplete responses), 5462 (67.2%) validated responses were obtained and used in the analyses.

### 2.3. Measurements

#### 2.3.1. Social-Environmental and Behavioral Risk Factors

Three indicators were used to measure each factor. Measurements for social-environmental factors were monthly household income, education level, and region. Alcohol use, tobacco use, and physical activity level were used for measuring behavioral risk factors. Since the KNHANES employed the second version of the Global Physical Activity Questionnaire (GPAQ) as the physical activity measurement item, total physical activity Metabolic Equivalent (MET) was calculated following the GPAQ analysis guide provided by the WHO [39]. Overall, a higher score indicated a higher social-environmental status and positive behaviors, while a lower score indicated the opposite. A detailed summary of the factors is presented in Table 1.

#### 2.3.2. Non-Communicable Diseases

With regard to data availability, NCDs, excluding diabetes, were categorized based on self-reports. Accordingly, those who reported having been diagnosed with MI, angina, stroke, and cancer were categorized as having the corresponding diseases (*yes: 0, no: 1*). Cancers were first divided into general cancers and female cancers. Gastric, liver, colon, lung, and thyroid cancers composed the cancer model. We considered breast and cervical cancers as exclusive to females and investigated them within the female responses dataset. In the case of diabetes, respondents who have taken oral hypoglycemic drugs or insulin injections or have fasting blood glucose higher than 126 mg/dL were categorized to have diabetes.

### 2.4. Data Analyses

To develop a comprehensive index of the factors for statistical analyses, each factor’s ratio index was calculated based on the following process.

First, the scores were assigned for each component of both social-environmental and behavioral risk factors. A score of one point was given to the lowest level of each indicator, and a point was added as the level went up. For example, the first quartile (Q1), the lowest level of household income, was assigned a score of one, while the fourth quartile (Q4), the highest level, was assigned a score of four. The scores for the indicators with a binary scale (e.g., region, alcohol use, and tobacco use) were adjusted to the highest scores in each factor. Between the two categories, one that represents undesirable status (e.g., current or former use of alcohol) was allocated a lower score than the rest. The scores for each component are given in Appendix A (Table A1).

**Figure A1:**
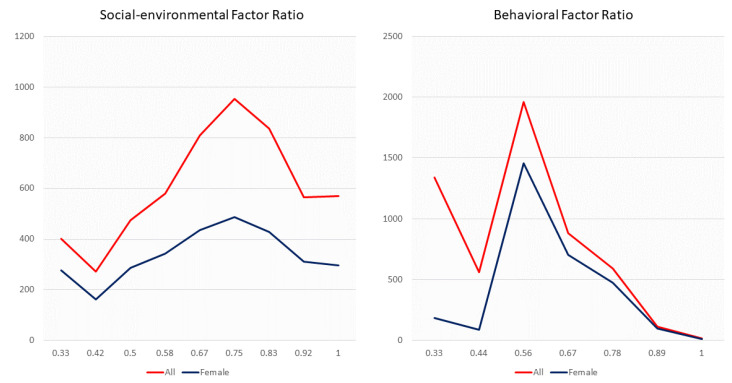
The distribution of the ratio scores.

Next, we calculated the ratio index by dividing the total score by the perfect score of each factor. If a participant is in the second quartile of income, the score was 2; and if a high school graduate, the score was 3. Whereas if the participant lived in the metropolitan city, the score totaled 4, thus the social-environmental factor index of this person was 0.75. The distributions of the ratio scores are presented in Appendix A (Figure A1).

Each NCD was analyzed based on the research model. The research model examines the effects of social-environmental factors on NCDs (H1) and behavioral risk factors (H3) as well as the effect of behavioral risk factors on NCDs (H2).

A series of path analysis was employed for stroke, MI, angina, diabetes, cancers, breast cancer, and cervical cancer with AMOS 18.0.

## 3. Results

### 3.1. Research Model Results

Table 2 shows the results of the research model. Social-environmental factors were significantly related to the prevalence of stroke (β = 0.101, *p* < 0.001), MI (β = 0.058, *p* < 0.001), angina (β = 0.077, *p* < 0.001), diabetes (β = 0.201, *p* < 0.001), and cancers (β = 0.069, *p* < 0.001), which include gastric, colon, lung, and thyroid cancers. However, there was no significant relationship between social-environmental factors and breast and cervical cancers. Analysis on the second hypothesis showed that behavioral risk factors significantly affect stroke (β = 0.031, *p* = 0.045), MI (β = 0.030, *p* = 0.046), angina (β = 0.039, *p* = 0.009), and diabetes (β = 0.049, *p* < 0.001). Finally, the significant negative effect of social-environmental factors on behavioral risk factors was observed in female cancer models (β = −0.053, *p* = 0.004), while no significant effect was found in models with a complete dataset (β = −0.024, *p* = 0.08).

### 3.2. Supplemental Analyses

Further, we conducted path analyses to comprehensively examine each factor’s effects by adding age and gender in the analyses. The supplemental analyses indicated that the models’ overall validation effects were decreased. However, there were no notable changes in tendencies.

## 4. Discussion

The current study investigated the effects of social-environmental and behavioral risk factors on NCDs using the national healthcare survey in South Korea. A series of path analyses were employed to examine the research model developed based on previous healthcare and NCD studies [17,18,19].

As indicated by the results, the effects of social-environmental factors on NCDs were validated. Thus, Hypothesis 1 was supported for stroke, MI, angina, diabetes, and cancers. These results support the previous literature that presents the causal linkage between social contexts and health outcomes [13,14,15]. Furthermore, Hypothesis 2 explored the relationship between behavioral risk factors and NCDs and was validated for stroke, MI, angina, and diabetes. Our findings suggest that social-environmental and behavioral risk factors are significantly correlated with the development of most NCDs, including cardiovascular diseases, diabetes, and several cancers. The results are consistent with the findings of earlier studies that focused on the effects of each risk factor on NCDs [18,21,25,27,32].

Regarding cancer, behavioral risk factors did not have notable effects. Following the findings of earlier studies that addressed the impact of behavioral risk factors on each cancer [25,40], the current study also confirmed the relationship between behavioral risk factors and cancers. For instance, Coups and Ostroff (2005) examined US national survey data and found that the effects of behavioral risk factors differ according to cancer types [40].

Social-environmental factors did not significantly affect behaviors in stroke, MI, angina, diabetes, and cancers models. Considering the strong relationship between social-environmental factors and behaviors suggested by preceding literature [13,14,33,34], the results possibly indicate that social-environmental factors other than income and education would affect the behaviors. Another possible interpretation is that scoring, which was an attempt to develop the comprehensive indices for each factor, might have diluted the intensity of the relationship.

Interestingly, social-environmental factors have a negative effect on behavioral risk factors in female cancer models. This result implies the possibility of the gender effects on the relationship between social-environmental factors and behavioral factors, as the earlier studies indicated [41,42].

From a practical perspective, the current study sheds light on the potentiality of using the national healthcare survey to address several risk factors and NCDs. Moreover, it employs a comprehensive view of social-environmental and behavioral risk factors as antecedents of NCDs. By integrating social-environmental and behavioral risk factors in a unified model, this study adds to discovering the sequential relationships of the factors.

Additionally, as an individual’s health status is one of the most complicated concepts in healthcare and medical areas, our findings can provide a better understanding of the antecedents of NCDs in South Korea. This approach allows us profound knowledge of NCDs since measuring an individual’s health status is a complex task that requires a variety of factors to be concerned [7,43].

Furthermore, as the current study employs the national healthcare survey, the findings can be easily generalized in South Korea. However, because each country has different social, economic, and cultural backgrounds, it is necessary to consider national healthcare policies [7]. Moreover, various risk factors should be considered when investigating NCDs from a national perspective.

## 5. Conclusions

In conclusion, the effects of social-environmental and behavioral risk factors on NCDs vary across the different types of diseases. The impacts of social-environmental factors on NCDs were significant in most of the NCDs examined but not in female cancers. Behavioral risk factors affect NCDs in similar patterns with social-environmental elements, but the effects were smaller than the previous factors. The relationship between social-environmental and behavioral risk factors was supported only in female cancer, which implies the gender effect. This study suggests including various preceding factors when investigating the national health status, which provides further understanding of the diseases.

## 6. Limitations and Future Research

Even though the current study provides several notable implications, some limitations remain. First, there may be other dominant factors that have significant effects on the development of NCDs [44]. This study did not adjust for gender, age, and diet, which have shown effects on NCDs in several studies that used larger datasets [45,46,47,48,49]. Nevertheless, some studies included the factors in the analyses with relatively smaller datasets [50,51,52]. Therefore, future studies should consider adjusting for the variables by adopting a larger dataset. Second, the current study examined data from the annual national survey of South Korea. A long-term follow-up time-series survey can provide a better understanding of NCDs from a national perspective. Furthermore, as the original data provide self-reports on the NCDs diagnosis except for diabetes, there is a possibility of inaccuracies in categorizing the diseases due to the recall bias. Next, even though the comprehensive approach can give benefits stated in the discussion section, the process of scoring might have attenuated the effects of each component. Lastly, there is a chance of bias due to the list-wise deletion method used in the data filtering process. Using more robust techniques could improve this issue. Future research should address these limitations to the extent of this stream of research.

## Figures and Tables

**Table 1:** Characteristics of the sample.

Characteristics	n (%)	Characteristics	n (%)
Age		Social-environmental factors	
19–30	726 (13.3)	Household income	
31–40	866 (15.9)	Q1	1018 (18.6)
41–50	1022 (18.7)	Q2	1303 (23.9)
51–60	1093 (20)	Q3	1515 (27.7)
61–70	963 (17.6)	Q4	1626 (29.8)
71–80	792 (1.5)	Education level	
Gender		Elementary school or less	1108 (20.3)
Men	2438 (44.6)	Middle school	548 (10.0)
Women	3024 (55.4)	High school	1686 (30.9)
Behavioral risk factors		University or more	2120 (38.8)
Alcohol use		Region	
Current or former use	4884 (89.4)	Non-metropolitan city	2961 (54.2)
Never use	578 (10.6)	Metropolitan city	3305 (45.8)
Tobacco use			
Current or former smoker	2157 (39.5)		
Non-smoker	3305 (60.5)		
Physical activity (MET level)			
Low	3487 (63.8)		
Moderate	1554 (28.5)		
High	421 (7.7)		

**Table 2:** The results of the research models.

Hypotheses	Standardized Coefficient (β)	Standard Error	Critical Ratio	Results
H1. Social-environment→ Stroke	0.101 ***	0.012	6.482	Supported
H2. Behavior→ Stroke	0.031*	0.014	2.005	Supported
H1. Social-environment→ MI	0.058 ***	0.008	3.941	Supported
H2. Behavior→ MI	0.030 *	0.010	1.995	Supported
H1. Social-environment→ Angina	0.077 ***	0.011	5.252	Supported
H2. Behavior→ Angina	0.039 **	0.013	2.614	Supported
H1. Social-environment→ Diabetes	0.201 ***	0.025	13.952	Supported
H2. Behavior→ Diabetes	0.049 ***	0.031	3.375	Supported
H1. Social-environment→ Cancers	0.069 ***	0.013	4.606	Supported
H2. Behavior→ Cancers	−0.006	0.015	−0.448	Not supported
H1. Social-environment→ Breast cancer	0.018	0.009	1.104	Not supported
H2. Behavior→ Breast cancer	−0.030	0.016	−1.750	Not supported
H1. Social-environment→ Cervical cancer	0.009	0.010	0.470	Not supported
H2. Behavior→ Cervical cancer	0.017	0.014	0.979	Not supported
H3. Social-environment→ Behavior ${}^{a}$	−0.024	0.011	−1.740	Not supported
H3. Social-environment→ Behavior ${}^{b}$	−0.053 **	0.012	−2.869	Supported

*** *p* < 0.001, ** *p* < 0.01, * *p* < 0.05. *^a^* Stroke, MI, angina, diabetes, and cancers model. *^b^* Breast cancer and cervical cancer model.

## Data Availability

The data that support the findings of this study are publicly available on the KNHANES website, https://knhanes.cdc.go.kr/knhanes/eng/index.do.

## Abbreviations

The following abbreviations are used in this manuscript:

NCD	Non-Communicable Disease
KNHANES	Korea National Health and Nutrition Examination Survey
WHO	World Health Organization
CVD	Cardiovascular Disease
MI	Myocardial Infarction
US	United States
KCDC	Korea Centers for Disease Control and Prevention
GPAQ	Global Physical Activity Questionnaire
MET	Metabolic Equivalent
GFI	Goodness of Fit Index
AGFI	Adjusted Goodness of Fit Index
RMSEA	Root Mean Square Error of Approximation

## Appendix A

**Table A1:** Scores for each factor.

Factors	Scores	Factors	Scores
Social-environmental factors		Behavioral risk factors	
Household income		Alcohol use	
Q1	1	Current or former use	1
Q2	2	Never use	3
Q3	3	Tobacco use	
Q4	4	Current or former smoker	1
Education level		Non-smoker	3
Elementary school or less	1	Physical activity (MET level)	
Middle school	2	Low	1
High school	3	Moderate	2
University or more	4	High	3
Region			
Non-metropolitan city	2		
Metropolitan city	4		
